# Identification and characterization of two classes of G1 β-bulge

**DOI:** 10.1107/S2059798320015533

**Published:** 2021-01-26

**Authors:** David P. Leader, E. James Milner-White

**Affiliations:** a College of Medical, Veterinary and Life Sciences, University of Glasgow, Glasgow G12 8QQ, United Kingdom

**Keywords:** β-bulge, β-bulge loop, β-link, protein motif

## Abstract

In standard β-bulges, a residue in one strand of a β-sheet forms hydrogen bonds to two successive residues (‘1’ and ‘2’) of a second strand. Two categories, ‘classic’ and ‘G1’ β-bulges, are distinguished by their dihedral angles: 1,2-α_R_β_R_ (classic) or 1,2-α_L_β_R_ (G1). It had previously been observed that G1 β-bulges are most often found as components of two quite distinct composite structures, suggesting that a basis for further differentiation might exist. Here, it is shown that two subtypes of G1 β-bulges, G1α and G1β, may be distinguished by their conformation (α_R_ or β_R_) at residue ‘0’ of the second strand. β-Bulges that are constituents of the composite structure named the β-bulge loop are of the G1α type, whereas those that are constituents of the composite structure named β-link here are of the G1β type. A small proportion of G1β β-bulges, but not G1α β-bulges, occur in other contexts. There are distinctive differences in amino-acid composition and sequence pattern between these two types of G1 β-bulge which may have practical application in protein design.

## Abstract 2

G1 β-bulges have been resolved into two subtypes that are differentiated by the dihedral angles at the position N-terminal to the doubleton. They have different amino-acid preferences and are unique constituents of higher-order protein architecture.

## 1. Introduction   

The β-bulge was first described (Richardson *et al.*, 1978 ▸) as a small motif in which, in its commonest and standard form, a residue (‘X’) in one antiparallel strand of a β-sheet forms main chain–main chain hydrogen bonds to two successive residues (‘1’ and ‘2’) of a second strand instead of making both hydrogen bonds to a single residue. This disrupts the regular β-sheet so that a bulge occurs, in some cases ending the participation of one or both of the strands in the sheet. Originally, two main types of β-bulge were distinguished: the classic β-bulge, with an α_R_ conformation at position 1, and the G1 β-bulge, with an α_L_ conformation at position 1 (Richardson *et al.*, 1978 ▸). (The definitions of α_R_, α_L_ *etc*. used here can be found in Section 5.) The name G1 derives from the frequent, but not invariable (Chan *et al.*, 1993 ▸), occurrence of glycine at this position. Other variants (‘wide’, ‘bent’ and ‘special’) have been described (Chan *et al.*, 1993 ▸) but are much less frequent, at only 10% of all β-bulges (Craveur *et al.*, 2013 ▸).

There has recently been renewed interest in β-bulges because the inclusion of both classic (Marcos *et al.*, 2017 ▸) and G1 (Dou *et al.*, 2018 ▸) β-bulges in protein design has proved to be necessary to achieve certain structural features. It was originally observed that G1 β-bulges occurred in the context of two quite different composite structures: the β-bulge loop (Milner-White, 1987 ▸) and what we call the β-link [a structure incorporating a β-bulge and a type II β-turn (Venkatachalam, 1968 ▸) directed away from the β-sheet (Richardson *et al.*, 1978 ▸)]. The question arises whether features of G1 β-bulges exist that favour the formation of one or the other of these composites and, if so, whether this information can be used in the design of synthetic proteins. We show here that by considering the conformation of the amino-acid residue N-terminal to the doubleton of the G1 β-bulge, such a distinction can be made.

## 2. Materials and methods   

This work employed two MySQL relational databases that modelled the atoms, residues and hydrogen bonds in different sets of proteins. The smaller one, Protein Motif, which was used in the initial phases of this work (Leader & Milner-White, 2009 ▸), contains information on 417 globular proteins from the 500 Protein Data Bank files from the Richardson laboratory (Lovell *et al.*, 2003 ▸). (Not all proteins in this and the larger data set were used because some contained duplicated amino-acid positions and other nonstandard features that conflicted with our database schema, causing them to be rejected.) Secondary-structure information and φ and ψ dihedral angles of residues were derived using *DSSP* (Kabsch & Sander, 1983 ▸), whereas for the χ and ω angles we utilized *BBDEP* (Dunbrack & Karplus, 1993 ▸). Backbone and inter-residue hydrogen bonds were derived using *HBPlus* (McDonald & Thornton, 1994 ▸).

The Protein Motif database was populated with a range of motifs derived from SQL queries specifying residue numbers and identities, dihedral angles and hydrogen bonds. For β-bulges the initial specification for the query was two consecutive residues (1 and 2) with a hydrogen bond between the main-chain CO of residue 1 and the main-chain NH of a third residue (X) and a hydrogen bond between the main-chain NH of residue 2 and the main-chain CO of residue X. A further stipulation was that residue 2 should have the β_R_ conformation (defined in Section 5). These β-bulges were divided into two classes: 1,2-α_R_β_R_ (classic) and 1,2-α_L_β_R_ (G1).

This database is part of the public web application *Motivated Proteins* (Leader & Milner-White, 2009 ▸) incorporating the molecular viewer *Jmol* (Herráez, 2006 ▸) and is also part of the desktop application *Structure Motivator* (Leader & Milner-White, 2012 ▸). *Motivated Proteins* allows the visualization of individual motifs in the context of the protein, whereas *Structure Motivator* allows the visualization of dihedral angles at different motif positions.

The second, larger, database, Proteins4K, was constructed specifically for this work. It contains information on 4485 globular proteins from the ‘Top 8000’ filtered structures from the Richardson laboratory (http://kinemage.biochem.duke.edu/databases/top8000.php). It was built using the same pipeline as Protein Motif, except that a script, *dihedral.pl*, kindly provided by Roland Dunbrack, was used instead of *BBDEP*. We used Proteins4K for command-line queries and populated it with β-bulges and the composite motifs encompassing them: β-bulge loops and β-links. The SQL queries for β-bulges made the same hydrogen-bond specifications as above, with the inclusion of dihedral angles at positions 0, 1 and 2 to provide subclasses.

Our approach differs from others employed to study structural motifs such as the *PROMOTIF* program (Hutchinson & Thornton, 1996 ▸). Although computationally less powerful than dedicated programs written in a language such as Fortran, SQL queries of a relational database modelling protein structure were used because of their flexibility. Regardless of the motifs that already populate the database, one can quickly retrieve and visualize information about constructs that suggest themselves in the course of an investigation.

## 3. Results and discussion   

### 3.1. Differentiation between the G1 β-bulges in β-bulge loops and β-links   

**Figure 1:**
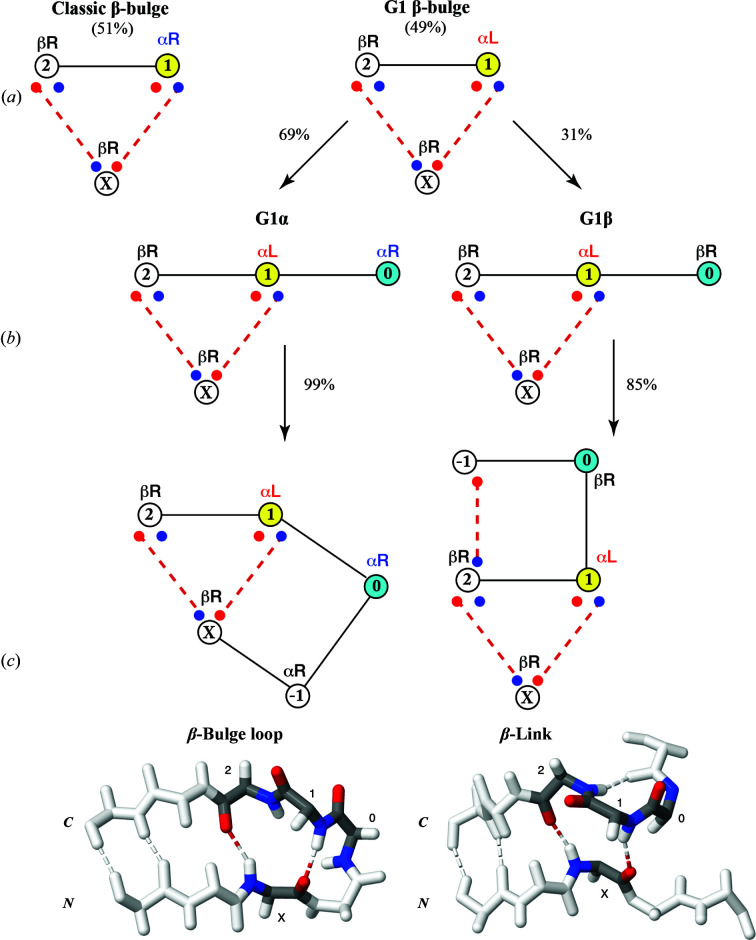
The different types of β-bulges and their relationship to composite structures. The singleton is designated ‘X’ and the doubleton residues as ‘1’ and ‘2’ in the N to C direction. In the diagrams, inter-main-chain hydrogen bonds are represented as broken lines, with the red circles representing O atoms and the blue circles representing N atoms. (*a*) Differentiation of standard and G1 β-bulges on the basis of their conformation at position 1 (yellow). (*b*) Subclassification of G1 β-bulges into types G1α and G1β on the basis of their conformation at position 0 (light blue). (*c*) Relationship of G1 β-bulges to larger composite structures: the β-bulge loop-5 and the β-link. Representative backbone three-dimensional structures of the composites in the context of two β-strands are shown below the diagrams, with the four residues of the β-bulge indicated in CPK colours and other residues in white: β-bulge loop (PDB entry 1a2p; Martin *et al.*, 1999 ▸) and β-link (PDB entry 2sak; Rabijns *et al.*, 1997 ▸).

**Figure 2:**
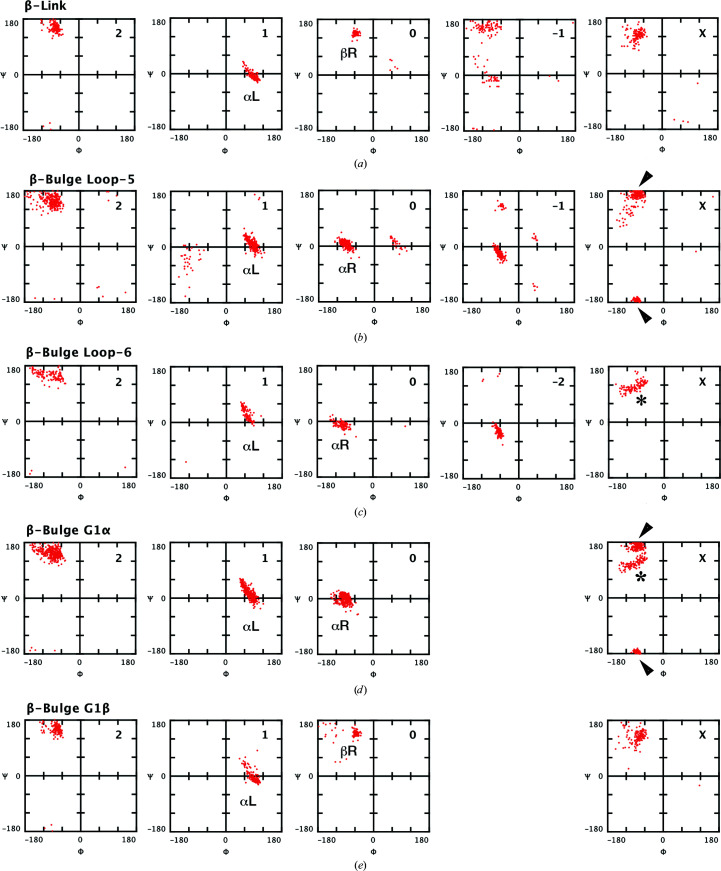
Main-chain dihedral angles at different positions of G1 β-bulges and composite structures containing them. The results are for the motifs present in the Protein Motif data set of 417 proteins visualized with *Structure Motivator*. The numbers at the top right of each frame indicate the residue position in the nomenclature of Fig. 1 ▸. (Position −1 of β-bulge loop-6 is not shown.)

The relational database of protein structural information, Protein Motif (Leader & Milner-White, 2009 ▸; see Section 2), containing 417 proteins was used for our initial work and for that in Fig. 2. In addition to primary data, it is populated with derived small structural motifs, including the β-bulge loop (Milner-White, 1987 ▸) and the β-link. The latter is a composite of a β-bulge and a type II β-turn where the 1,2-positions of the β-bulge constitute the 3,4-positions of the β-turn (Fig. 1 ▸). [The β-link was originally described by Richardson *et al.* (1978 ▸), but was not named by them and has been somewhat neglected until recently.]

While visualizing the dihedral angles of β-bulge loops and β-links as Ramachandran plots in the desktop application *Structure Motivator* (Leader & Milner-White, 2012 ▸), it became evident that the G1 β-bulges belonging to these two composite motifs differed at what would be position ‘0’, N-terminal to the doubleton. In the β-bulge loop this had the α_R_ conformation, whereas in the β-link it had the β_R_ conformation. When modified versions of β-bulges, extended to include position ‘0’, were viewed in the *Structure Motivator* application two separate distributions of dihedral angles were apparent (Fig. 2 ▸).

We have therefore altered the definition of the β-bulge to include position ‘0’ and have subdivided the G1 β-bulges into two classes: G1α, 0,1,2-α_R_α_L_β_R_, and G1β, 0,1,2-β_R_α_L_β_R_. These are illustrated diagrammatically in Fig. 1 ▸(*b*). Fig. 1 ▸(*c*) shows examples of the two composite motifs within protein structures.

### 3.2. Occurrence of G1 β-bulges outside β-bulge loops or β-links   

Having established that the extended definition of G1 β-bulges allows one to distinguish those present in β-bulge loops from those in β-links, it was pertinent to ask whether β-bulges occurred in other contexts than within these composites. We performed the following analysis using the tenfold larger database Proteins4K. We first queried the database for all β-bulges conforming to the pattern 0,1,2-θα_R_β_R_ (classic) or 0,1,2-θα_L_β_R_ (G1), stipulating that the pattern of hydrogen bonding to residue X be as in Fig. 1 ▸(*a*). (θ represents any of the four pairs of dihedral angles, α_L_, α_R_, β_L_ and β_R_.) The number of instances of each of the eight subtypes so defined are given in the ‘Total’ column of Table 1 ▸. It can be seen that almost all classic β-bulges are of subtype 0,1,2-β_R_α_R_β_R_ and that the vast majority of G1 β-bulges are of the subtypes G1α or G1β. (The proportions of these three types are included in Fig. 1 ▸.) As can be seen in Fig. 2 ▸, for any specification such as β_R_, the values of the dihedral angles found at different positions and in different motifs vary. Mean values for the major types of β-bulge are given in Supplementary Table S1.

The Proteins4K database was populated with these subclasses of β-bulges, which were then queried to determine the proportion in higher-order structures. To identify β-bulges in loops such as the β-bulge loop-5 (Fig. 1 ▸ *c*), loop-6 or higher, the query was for the position of residue X relative to residue 1. To identify β-bulges in β-links the query was for a hydrogen bond between positions −1 and 2 of the β-bulge (Fig. 1 ▸ *c*). The final two columns of Table 1 ▸ show that 99% of G1α β-bulges occur in β-bulge loops and 85% of G1β β-bulges occur in β-links. The 15% of G1β β-bulges that are not in β-links are considered below.

### 3.3. Amino-acid preferences of G1α and G1β β-bulges   

**Figure 3:**
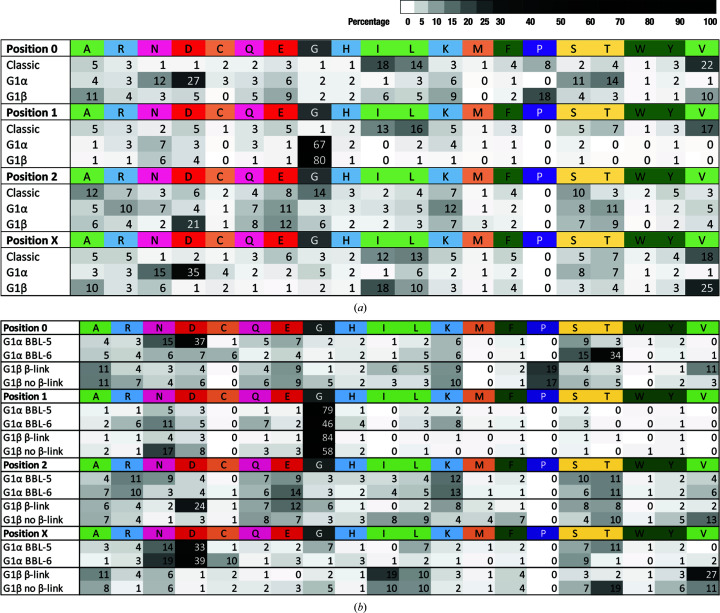
Amino-acid compositions of different classes of β-bulge in the Proteins4K database. The four positions 0, 1, 2, X are as shown in Fig. 1 ▸. The numbers in the figure are the percentages of the total for all 20 contributed by each individual amino-acid residue. (*a*) The three classes of β-bulge: classic (5133), G1α (3348) and G1β (1506). (*b*) Division of G1α β-bulges into those present in β-bulge loop-5 (BBL-5; 2154) and loop-6 (BBL-6; 1155) and of G1β β-­bulges into those present in (1283) or absent from (223) β-links.

Fig. 3 ▸ compares the amino-acid compositions of the main types of β-bulge at the four defining positions, 0, 1, 2 and X. Fig. 3 ▸(*a*) shows that although both G1α and G1β β-bulges have a high proportion of glycine at position 1, their amino-acid compositions differ considerably at the other positions. This is most marked for position X, where G1α β-bulges are rich in amino acids with side chains that have hydrogen-bonding potential (50% Asn/Asp, 15% Ser/Thr), whereas G1β β-bulges are rich in aliphatic amino acids at this position (63% Ala/Ile/Leu/Val). Position 0, the conformation of which differentiates the G1 β-bulges, also shows differences in amino-acid composition, with G1α β-bulges being rich in residues with polar side chains (73% Asn/Asp/Gln/Glu/Ser/Thr), whereas G1β β-bulges have 48% Ala/Lys/Pro/Val. A degree of similarity occurs at position 2, with both types of G1 β-bulge having many residues with polar side chains, although G1β β-bulges are enriched in aspartate (G1β, 21%; G1α, 4%).

We separated G1α β-bulges into those that are components of β-bulge loop-5 and β-bulge loop-6 structures, and separated G1β β-bulges into those that are components of β-links and the 15% that are not. Their amino-acid compositions are shown in Fig. 3 ▸(*b*). It is evident that G1α β-bulges belonging to β-bulge loop-5 motifs have a higher proportion of glycine residues at position 1 than those in β-bulge loop-6 motifs, and that at position 0 their polar amino acids are skewed to aspartate and asparagine at the expense of threonine. G1β β-bulges within β-links are likewise enriched in glycine at position 1 compared with those not in β-links. Also noteworthy is that the enrichment in aspartate at position 2 of G1β β-bulges is confined to those in β-links.

**Figure 4:**
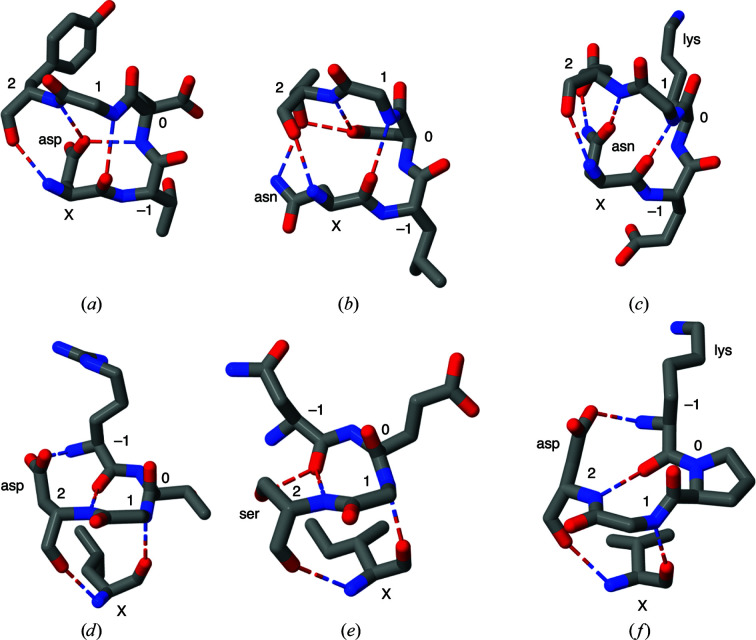
Examples of additional hydrogen bonding in composite structures incorporating G1 β-bulges: (*a*) G1α β-bulge loop (PDB entry 119l, residues 20–24; Blaber *et al.*, 1993 ▸), (*b*) G1α β-bulge loop (PDB entry 1aqb, residues 124–128; Zanotti *et al.*, 1998 ▸), (*c*) G1α β-bulge loop (PDB entry 1fnc, residues 239–243; Bruns & Karplus, 1995 ▸), (*d*) G1β β-link (PDB entry 1a62, residues 55, 92–95; Allison *et al.*, 1998 ▸), (*e*) G1β β-link (PDB entry 1k7i, residues 318–321, 334; Hege & Baumann, 2001 ▸), (*f*) G1β β-­link (PDB entry 1fdr, residues 9, 83–86; Ingelman *et al.*, 1997 ▸).

Some of these differences in amino-acid composition can be rationalized in terms of constraints imposed by the composite structures of which G1 β-bulges are components. This is illustrated in Fig. 4 ▸. The polar side chain at position X of approximately 70% of G1α β-bulges (which is rare at this position in G1β β-bulges) may be involved in either backbone (Fig. 4 ▸ *a*) or side-chain (Figs. 4 ▸ *b* and 4 ▸ *c*) hydrogen bonding within the β-bulge loop. In the case of G1β β-bulges additional side-chain hydrogen bonding is often found from a polar side chain at position 2 to the backbone NH or CO at position −1 (Figs. 4 ▸ *d*, 4 ▸ *e* and 4 ▸ *f*). The amino-acid residue frequently involved is aspartate, which is much less abundant in G1β β-bulges that are not parts of β-links. Aspartate is equally rare at this position in G1α β-bulges. Hydrogen bonding by aspartate and asparagine side chains to nearby main-chain atoms has previously been observed in small motifs (Eswar & Ramakrishnan, 1999 ▸; Wan & Milner-White, 1999 ▸; Duddy *et al.*, 2004 ▸). The greater abundance of glycine residues at position 1 in G1 β-bulges in the more tightly constrained β-bulge loop-5 motifs and β-links suggests a role in stabilizing the respective β-turns in these latter structures. It should also be mentioned that there is a clear difference in the distribution of dihedral angles found at position X of β-bulge loop-5 and β-bulge loop-6 motifs within the general β_R_ region, as indicated by arrowheads and asterisks, respectively, in Fig. 2 ▸.

### 3.4. Sequence patterns and heterogeneity of G1 β-bulges   

The difference in the dihedral angles of G1α and G1β β-bulges enables one to distinguish them in proteins of known three-dimensional structure. In a similar way, a machine-learning approach allows one to assign the most probable structure of the two on the basis of amino-acid preferences (D. P. Leader, E. J. Milner-White & S. Rogers, unpublished work). However, in engineering proteins with specific subtypes of β-bulge a sequence of amino acids must be selected that is likely to produce the desired structure: a choice made from the many combinations of the most frequent amino acids in the four positions 0, 1, 2 and X.

Supplementary Table S2 contains a list of sequence patterns for the G1 β-bulges. Although the number of variants is large, it is instructive to examine the five that occur most frequently in each category, as shown in Table 2 ▸. For G1α β-bulges present in β-bulge loop-5 motifs, tripeptides for the 0, 1, 2 sequence of the type DG(S/T/N) are common, as expected from the amino-acid composition, and allow the selection of combinations with residue X that are uncommon in other subtypes. The frequent occurrence of the 0, 1, 2, X combination KGEN is less expected: it is as abundant as all other –GEN combinations in total. Its structure is shown in Fig. 4 ▸(*c*), with hydrogen bonds between the asparagine side chain at position X and the glutamate side-chain O atom and backbone NH group. The lysine residue is oriented away from the β-bulge hydrogen bonds towards the surface of the protein and, in all instances except one, does not interact with the carboxyl group of the glutamate. For the G1α β-bulges in β-bulge loop-6 motifs the most common sequences are consistent with the frequencies of amino acids.

The situation for the majority of G1β β-bulges, those that form β-links, is that the most abundant combinations 0, 1, 2, X are of the type –DGV, as in the amino-acid compositions. The most frequent sequence pattern is PGDV, a reflection of proline being most frequent at position 0. What is not evident from Table 2 ▸ is that the amino acid at position −1 is either lysine or arginine in half of the 27 instances. The disposition of these side chains is towards the surface of the protein away from the β-bulge hydrogen bonds (Fig. 4 ▸ *f*), resembling that of lysine at position 0 in the KGEN motif of G1α β-bulges. In this case, however, about half of the basic side chains interact with the carboxylate group of the aspartate. These observations are consistent with previous analysis of the distribution of amino acids in β-sheets, which showed that lysine and arginine are often found at the edges of sheets (Fujiwara *et al.*, 2014 ▸), where most G1 β-bulges are located.

Although we believe that this analysis of sequence patterns will be useful in protein design, it is evident that other factors determine whether a particular pattern will be appropriate in any instance.

## 4. Conclusions   

This work answers a longstanding question about G1 β-bulges by showing that there are two subtypes, G1α and G1β, which can be differentiated on the basis of the conformation at position 0. A reclassification of β-bulges on this basis has been implemented in the Protein Motif database and the publicly available web (Leader & Milner-White, 2009 ▸) and desktop (Leader & Milner-White, 2012 ▸) applications that incorporate it.

An important aspect of this reclassification is that these two types of G1 β-bulge are integral components of two different composite structures: G1α β-bulges in β-bulge loops and G1β β-bulges in β-links. G1α β-bulges and the loops containing them occur in different types of β-sheet as an alternative to the simple β-turn in β-hairpin and β-meander structures. In β-barrels, these loops may serve to reduce strain (Dou *et al.*, 2018 ▸). The β-links (Richardson *et al.*, 1978 ▸), in which the majority of G1β β-bulges reside, have received less attention, but our unpublished work shows that they are important in small β-barrels and in β-sandwich proteins. The analysis of G1 β-bulges in the present work should help to inform the design of engineered proteins in these categories.

## 5. Abbreviations   

α_R_ encompasses the range of dihedral angles −140° < φ < −20°, −90° < ψ < 40°, α_L_ the range 20° < φ < 140°, −40° < ψ < 90°, β_R_ the range 150° < φ or φ < −25°, 40° < ψ or ψ < −150° and β_L_ the range 20° < φ < 140°, −180° < ψ < −80° (here the γ_L_ region is included within the α_L_ region). These abbreviations are used in shorthand descriptions of β-bulges to indicate the conformations at residues 0, 1 and 2 on the ‘bulged’ strand: for example, 0,1,2-α_R_α_L_β_R_ indicates a β-bulge in which residue 0 has the α_R_ conformation, residue 1 has the α_L_ conformation and residue 2 has the β_R_ conformation.

## Supplementary Material

Supplementary Table S1. DOI: 10.1107/S2059798320015533/jb5022sup1.pdf

Supplementary Table S2. DOI: 10.1107/S2059798320015533/jb5022sup2.xlsx

## Figures and Tables

**Table 1:** Occurrence of different types of β-bulge and their participation in composite motifs Standard β-bulges were retrieved from a database of 4485 proteins by queries specifying the hydrogen-bonding pattern in Fig. 1 ▸(*a*) and the dihedral angles given in the Subtype column. Queries were made to determine the number of each subtype present in the two composite motifs indicated. For β-bulge loops this involved the additional specification that the singleton residue X was at position −2, −3 or −4 for β-bulge loop-5, loop-6 or loop-7, respectively. For β-­links this involved the additional specification of a hydrogen bond between the peptide-bond O atom at position −1 and the peptide-bond N atom at position 2 in the numbering of Fig. 1 ▸(*c*).

Type	Subtype	Total	β-Bulge loop	β-Link
1,2-α_R_β_R_ (classic)	0,1,2-α_R_α_R_β_R_	38 (<1%)	25†	0
	0,1,2-β_R_α_R_β_R_	5133 (50%)	2	0
	0,1,2-α_L_α_R_β_R_	116 (1%)	101‡	0
	0,1,2-β_L_α_R_β_R_	8 (<1%)	0	2
1,2-α_L_β_R_ (G1)	0,1,2-α_R_α_L_β_R_ (G1α)	3348 (33%)	3312§	0
	0,1,2-β_R_α_L_β_R_ (G1β)	1506 (15%)	2	1283
	0,1,2-α_L_α_L_β_R_	128 (1%)	123¶	68
	0,1,2-β_L_α_L_β_R_	13 (<1%)	0	0

† β-Bulge loop-5 (24 instances), β-bulge loop-6 (one instance).

‡ β-Bulge loop-5 (88 instances), β-bulge loop-6 (13 instances).

§ β-Bulge loop-5 (2154 instances), β-bulge loop-6 (1155 instances), β-bulge loop-7 (three instances).

¶ β-Bulge loop-5 (81 instances), β-bulge loop-6 (8 instances), β-bulge loop-7 (33 instances).

**Table 2:** Sequence patterns for G1 β-bulges The five most frequently occurring patterns are shown for each motif. The frequency is per thousand motifs, with the actual number of instances in parentheses. Where no instance of a sequence pattern was found for a particular motif the entry in the table has been left blank to facilitate comparison.

Sequence	Motif			
(0, 1, 2, X)	G1α (BBL5)†	G1α (BBL6)‡	G1β (β-link)§	G1β (no β-link)¶
KGEN	11 (24)		2 (2)	
DGND	10 (22)			
DGTN	10 (21)	1 (1)		
DGSN	9 (20)	2 (2)		
DGTL	9 (19)			
TGED	1 (2)	16 (19)		
TGKD	2 (4)	16 (18)		
TGEN		10 (12)		
TGRD	0 (1)	10 (11)		
TGAD	1 (3)	9 (10)		
PGDV			21 (27)	
VGDV			12 (16)	
EGDV			9 (12)	
IGDV			9 (12)	
KGDV			9 (12)	
QNEL				13 (3)
ADVT				9 (2)
AGIT				9 (2)
AGVT				9 (2)
KDYY				9 (2)

† G1α β-bulge within a β-bulge loop-5 (1143 unique patterns in 2153 motif occurrences).

‡ G1α β-bulge within a β-bulge loop-6 (854 unique patterns in 1152 motif occurrences).

§ G1β β-bulge within a β-link (824 unique patterns in 1283 motif occurrences).

¶ G1β β-bulge not within a β-link (213 unique patterns in 223 motif occurrences).
