# Supplementary material for: Identification and characterization of two classes of G1 β-bulge
Source: Acta Crystallogr D Struct Biol. 2021 Jan 26;77(Pt 2):217–23. doi: 10.1107/S2059798320015533 (PMC7869896; doi:10.1107/S2059798320015533)
Supplement: Supplementary file 1 [file d-77-00217-sup1.pdf]

STRUCTURAL
BIOLOGY

Volume 76 (2020)

Supporting information for article:

Identification and characterization of two classes of G1 β-bulge

David P. Leader and James E. Milner-White

Table S1 Dihedral angles at different positions of the three main subtypes of β-bulge

	0		1		2		X	
	φ	ψ	φ	ψ	φ	ψ	φ	ψ
Classic	–82 ± 21	126 ± 23	–99 ± 16	–32 ± 16	–155 ± 13	151 ± 15	–113 ± 20	130 ± 13
G1α	–93 ± 14	–2 ± 13	79 ± 15	14 ± 19	–91 ± 25	145 ± 13	–87 ± 18	151 ± 28
G1β	–59 ± 17	134 ± 13	87 ± 12	–3 ± 17	–81 ± 20	140 ± 52	–80 ± 21	124 ± 20

The broad dihedral angle specifications for the selections were Classic — 0,1,2-$\beta_R\alpha_R\beta_R$, G1α — 0,1,2-$\alpha_R\alpha_L\beta_R$, G1β — 0,1,2-$\beta_R\alpha_L\beta_R$. Means and standard deviations are shown.
